# Primary Amoebic Meningoencephalitis in an Infant due to *Naegleria fowleri*

**DOI:** 10.1155/2011/782539

**Published:** 2011-11-03

**Authors:** Vinay Khanna, Ruchee Khanna, Shrikiran Hebbar, V. Shashidhar, Sunil Mundkar, Frenil Munim, Karthick Annamalai, Deepak Nayak, Chiranjay Mukhopadhayay

**Affiliations:** ^1^Department of Microbiology, Kasturba Medical College, Manipal, 576104 Karnataka, India; ^2^Department of Pathology, Kasturba Medical College, Manipal, 576104 Karnataka, India; ^3^Department of Paediatrics, Kasturba Medical College, Manipal, 576104 Karnataka, India

## Abstract

Primary amoebic meningoencephalitis (PAM) caused by free-living amebae *Naegleria fowleri* is a rare and fatal condition. A fatal case of primary amoebic meningoencephalitis was diagnosed in a 5-month-old infant who presented with the history of decrease breast feeding, fever, vomiting, and abnormal body movements. Trophozoites of *Naegleria fowleri* were detected in the direct microscopic examination of CSF and infant was put on amphotericin B and ceftazidime. Patient condition deteriorated, and he was discharged against medical advice and subsequently expired. We also reviewed previously reported 8 Indian cases of primary amoebic meningoencephalitis (PAM) and observed that for the last 5 years, none of the patients responded to amphotericin B. Has an era of amphotericin B-resistant *Naegleria fowleri* been emerged? Management strategy of PAM needs to be reviewed further.

## 1. Introduction

*Naegleria fowleri*, the cause of primary amoebic meningoencephalitis (PAM), is present throughout the world. The clinical course of *Naegleria *neurological infection has a sudden onset and a fulminant course due to diffuse hemorrhagic necrotizing meningoencephalitis. Rapid progression of the disease process and limited awareness among the clinicians and diagnostic staff make this disease a diagnostic challenge. Only 8 cases of PAM due to *Naegleria fowleri* have been reported from India to date to best of our knowledge [1–7]. We present a case of PAM in a 5-month-old child due to *Naegleria fowleri *and review the previous cases.

## 2. Case Report

**Figure 1:**
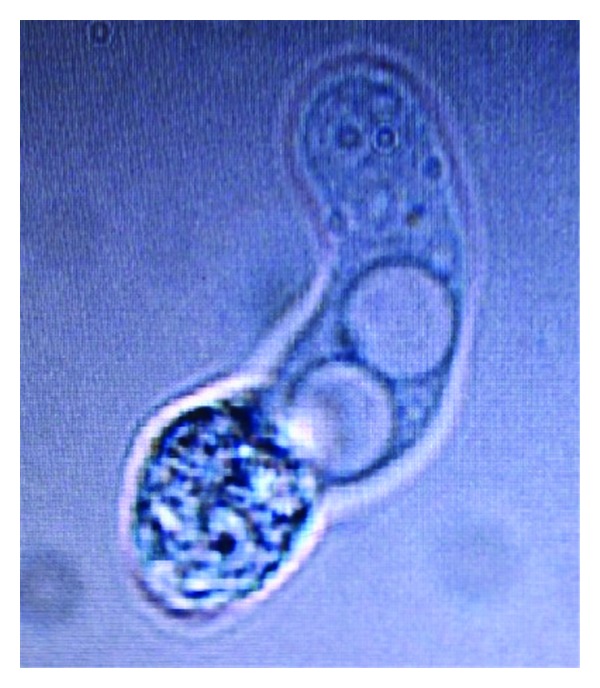
CSF methylene blue wet preparation showing *Naegleria fowleri* with granular cytoplasm and endosomes.

**Figure 2:**
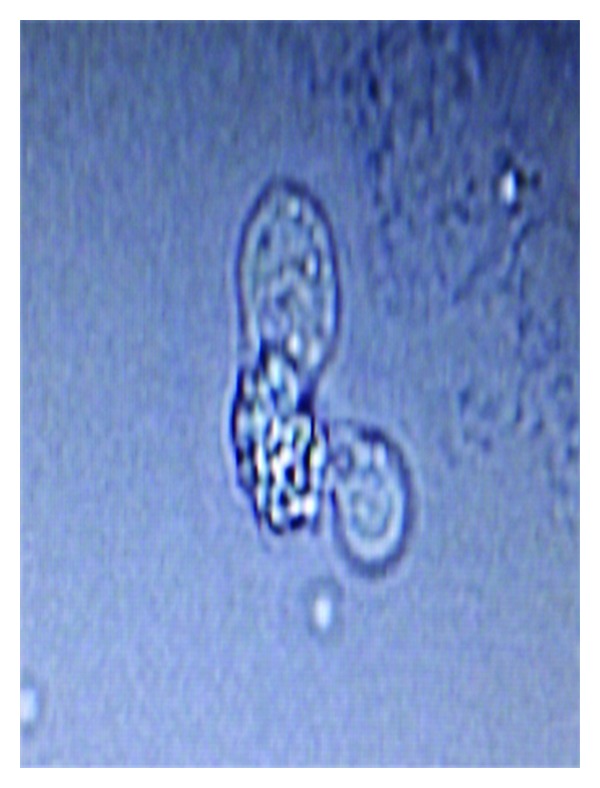
CSF normal saline mount showing *Naegleria fowleri* with characteristic lobopodia.

A 5-month-old infant was admitted to paediatric department with a two-day history of fever, decreases breast feeding, vomiting, and abnormal body movements. His birth history as well as developmental history was uneventful. The child was immunized up to date. The mother had no signs of mastitis.The child was apparently asymptomatic until two-days prior to admission, and then presented with decreased breast feeding, continuous high-grade fever, and two episodes of vomiting following semisolid feed which contained food particles which was neither bile nor blood stained. On the day of admission, mother noticed tonic-clonic movements which was limited to lower limb initially and gradually involved the whole body. After that episode, the child had continuous unsteadiness of trunk and neck. On admission, his weight was 6.2 kg, temperature 38°C, heart rate 140 beats/min, respiratory rate 60 breaths/min, blood pressure 106/70 mmHg, and SpO_2_ was 100% at room temperature. CNS examination revealed bulging and tensed anterior fontanelle with positive Kernig's sign and presence of nystagmus. White blood cell count was 17,700 cells/cumm with 89% polymorphonuclear cells. Provisional diagnosis of acute bacterial meningitis was made, and the child was put empirically on Inj. Ceftriaxone 250 mg TID and Inj. Amikacin 50 mg BD with anticonvulsants and antiedema measures. Lumbar puncture was done, and CSF was sent for microbiological and cytological analysis. CSF was clear, and biochemical analysis showed glucose concentration of 5 mg/dL (Normal value 40–85 mg/dL), proteins concentration of 731 mg/dL (Normal value 15–45 mg/dL), and chloride ions concentration of 105 mEq/mL. CSF counts revealed a total WBC count of 990/cumm (normal value < 5/cumm) with predominantly lymphocytes. No bacteria or fungal elements were seen on Gram stain. Bacterial culture was sterile. Microscopic examination of wet CSF preparation showed motile trophozoite of free living amoeba which was suggestive of *Naegleria fowleri *(Figures 1 and 2). Final diagnosis of PAM was made, and therapy with IV amphotericin B 3 mg and IV ceftazidime 300 mg was started, but his condition deteriorated and was taken home by his relatives in a moribund condition against medical advice and subsequently died.

## 3. Discussion

*Naegleria fowleri* is a thermophilic organism, and its distribution is limited by the sensitivity of the cysts to desiccation and warm environment for growth. Infection depends on the number of trophozoites and insufflation of water into the sinuses which is determined by the type of swimming and the concentration of organisms in the water. The route of invasion is almost certainly through the olfactory neuroepithelium [8]. Since most cases were diagnosed in children and young adults, individuals host susceptibility may be an important factor.

As shown in Table 1, eight cases, four of which had history of exposure to water. For the rest of the cases, sources could not be found. According to the literature review, most of the cases of PAM are usually misdiagnosed as acute bacterial meningitis or tubercular meningitis. The detection of amoeba in wet preparation of CSF using ordinary light microscopy or in counting chamber are valuable for correct diagnosis, but there are chances that these may sometimes be overlooked, as there is limited awareness and expertise. There are some limitation in this study, as CT scan of the brain was not done due to financial constrained although it should have been done in this case, as hydrocephalus, hemorrhagic edematous confluent foci, can increase intracranial pressure which might have been detected, and treatment could have been instituted in the form of external ventricular drainage. In our case, there was no history of contact with water. Inspite of that, we tried to look for the presence of amoeba in the well water as well as the tap water from the patient's house, but it was not significant.

Fulminant course of PAM demands a rapid diagnosis and wet preparation of CSF remains the main stay in resource-constrained countries like India. A triplex real-time TaqMan PCR assay for simultaneous identification of *Acanthamoeba spp., B. mandriallaris*, and *N. fowleri *has been developed, and it could be useful for fast laboratory diagnosis [9].

There is no gold standard treatment regimen for PAM. Amphotericin B, rifampicin, and sulphadiazine along with antiedema measures have been tried with limited success. A recent study has indicated that amphotericin B and azithromycin have a synergistic action against *N. fowleri*, suggesting that the combined use of these agents may be beneficial in treating PAM [10]. Combination of amphotericin B along with fluconazole and rifampicin has also been tried and has shown promising results [11].

According to the literature review, amphotericin B is the antinaeglerial agent for which there is evidence of clinical effectiveness when administered intravenously at high doses or intrathecally along with miconazole administered intravenously [12, 13]. We observed that in last 5 years, none of the cases reported from India improved inspite of starting amphotericin B. Amphotericin B-resistant strains of *Naegleria fowleri* are not discussed so far, and the possibility of resistant strains could not be ruled out.

## 4. Conclusion

PAM demands a special attention from clinicians for diagnosis and management. Though only nine cases are reported from our country, it may be a tip of an iceberg. Other cases may be misdiagnosed as bacterial meningitis or tubercular meningitis due to lack of awareness and may be associated with worst outcome. By analysing the cases reported from India in the last 5 years, we would like to raise an issue whether an era of amphotericin B-resistant *Naegleria fowleri *has been emerged. Is it a high time for an effective antinaeglerial agent to be discovered? Management strategy of PAM needs to be reviewed further.

## Figures and Tables

**Table 1:** Indian cases of *Naegleria fowleri* meningitis.

Year	Author	Age/sex	Contact with water	Investigation	Treatment	Outcome
1971	Pan and Ghosh [1]	3 yrs/male	Yes	CSF wet mount	Amphotericin B, Sulphadiazine, and Dexamethasone	Cured
1971	Pan and Ghosh [1]	5 months/male	—	CSF wet mount	Sulphadiazine, Streptomycin, and Amphotericin B	Cured
1998	Singh et al. [2]	8 yrs/male	No	CSF wet mount	Amphotericin B, and Rifampicin	Cured
2002	Shenoy et al. [3]	4 months and 8 days/male	Yes	CSF wet mount	Amphotericin B	Died
2002	Jain et al. [4]	26 yrs/female	No	CSF wet mount	Amphotericin B, and Rifampicin	Cured
2005	Hebbar et al. [5]	5 months/male	Yes	CSF wet mount	Amphotericin B, Chloramphenicol, and Metronidazole	Died
2006	Tungikar et al. [6]	30 yrs/male	No	CSF wet mount	Cefotaxime, Amikacin, Metronidazole, and Azithromycin	Died
2008	Kaushal et al. [7]	36 yrs/male	Yes	CSF wet mount	Amphotericin B, Rifampicin, and Ceftazidime	Died
Present case	Vinay et al.	5 months/male	—	CSF wet mount	Amphotericin B, and Ceftazidime	Died
